# Supplementary material for: Learning curve and surgical outcome of robotic assisted colorectal surgery with ERAS program
Source: Sci Rep. 2022 Nov 29;12:20566. doi: 10.1038/s41598-022-24665-w (PMC9709162; doi:10.1038/s41598-022-24665-w)
Supplement: Supplementary file 2 — Supplementary Information 2. [file 41598_2022_24665_MOESM2_ESM.zip › RAL Raw data-Ñ[▒K/048/Patho_ñw╝╨░O▒Kñσ.pdf]

臺中榮民總醫院 臺中市臺灣大道四段1650號 病理部活體組織切片 臨床醫學實驗及病理診斷中心 檢驗(查)報告

[redacted]

Pathologic diagnosis:
1. Rectum, robotic TME --- Status post neoadjuvant CCRT with extensive ulceration, acute and chronic inflammation and submucosal hemorrhage. No residual carcinoma found. The bilateral cut ends and perirectal lymph nodes (0/17) are free.
2. Rectum, cut end, distal, resection --- Chronic inflammation.
3. IMA root, dissection --- Unremarkable fibrovascular tissue and lymph nodes (0/5).

Ancillary study for diagnosis:
IHC stain for AE1/AE3 (no abnormal epithelium) done on section E.

Prognostic and predictive factor:
1. Tumor size: No residual carcinoma.
2. Depth of invasion: No evidence of residual tumor.
3. Histologic grade: Not applicable.
4. Angiolymphatic invasion: Not identified.
5. Perineural invasion: Not identified.
6. Tumor deposits (discontinuous extramural extension): Not identified.
7. Macroscopic tumor perforation: Not identified.
8. Surgical Margins: a) Proximal Margin: Uninvolved. b) Distal Margin: Uninvolved. c) Radial or Mesenteric Margin: Uninvolved.
9. Lymph node status: Uninvolved (0/22).
10. Extranodal involvement: Not identified.
11. Tumor regression grading S/P CCRT: Present, no viable cancer cells (complete response, score 0).
12. Type of polyp in which invasive carcinoma arose: Not identified.
13. Additional pathologic findings: Not identified.
14. Pathological TNM Stage: ypT0N0(According to the eighth edition, American Joint Committee on Cancer Staging Guidelines for Tumors).
15. TNM descriptors: y (Post-treatment).

Gross description:
The specimen consists of 1) a segment of colon, 14 cm in length and 3 cm in proximal cut end circumference, 4 cm in distal circumference, and perirectal soft tissue. A gray tan ulcerative lesion, 2.8x1.6 cm, is noted, 0.6 cm to the distal cut end. On cross section, submucosal fibrosis is noted and noo definite if=nfiltrating mass lesion is identified grossly. The both cut ends are free. 2) distal cut end, a piece of tan mucosa-covered soft tissue, 2.2x1.1x0.5 cm. 3) IMA root, a piece of tan yellow soft tissue, 2.3x1.2x0.7 cm. Representative part for section: A) proximal cut end B) distal cut end C-E) tumor F) colon G-H) specimen 2-3 J-K) lymph nodes.

Note:
This case has been peer reviewed by two doctors.
#T-59600_2 #M-38000_2 0 1105 SP0000
#T-C4560_2 #M-09183_2 0 1105 000000

已電子簽章

敬告：本單僅供醫師參考使用，不具任何申請效力！！
請病患妥為保管，若不慎遺失需自負個人資料外洩責任！
